# Effects of continuous positive airway pressure on respiratory mechanics and breathing pattern in healthy individuals

**DOI:** 10.1186/2197-425X-3-S1-A268

**Published:** 2015-10-01

**Authors:** E Soilemezi, E Koco, M Oikonomou, M Georgiadis, D Matamis

**Affiliations:** Papageorgiou General Hospital, Thessaloniki, Greece

## Introduction

Variable levels of positive end expiratory pressure (PEEP) are widely used during mechanical ventilation or during spontaneous breathing for acute or chronic lung diseases. In this study, respiratory indirect plethysmography (RIP), electromyography (EMG) of the transversus abdominis and scalene muscles and indirect calorimetry are used to achieve an integrated picture of the changes in the respiratory system after exposure to 10 cmH_2_O CPAP.

## Objectives

To investigate the changes in breathing pattern, respiratory muscle mechanics and work of breathing induced by 10 cmH_2_O of continuous positive airway pressure (CPAP).

## Methods

Fourteen young, healthy individuals participated in the study, assessed in the semi-recumbent position initially at ZEEP and then at 10 cmH_2_O CPAP. RIP was used to evaluate the contribution of the rib cage (RC) and abdominal (AB) compartments to the inspired tidal volume, and to allow measurement of the continuous degree of asynchrony between the thoracic and abdominal movements. EMG activity of the accessory respiratory muscles (transversus abdominis and scalene muscles) and measurements of oxygen consumption (V02) were recorded during both ZEEP and CPAP settings.

## Results

Tidal volume increased from ZEEP (738 ± 208 ml) to CPAP (896 ± 272 ml, p < 0,05) with RIP revealing that this increase was mainly due to RC rather than AB contribution. No significant thoraco-abdominal asynchrony was observed in the CPAP setting. During the first twenty breaths during CPAP the difference between mean tidal volume inspired (862 ± 235 ml) and mean tidal volume expired (831 ± 875 ml) was 31 ml, and the volume trapped was approximately 600 ml. This volume trapped was accommodated during the first four CPAP breaths, after which mean tidal volume inspired and mean tidal volume expired did not differ significantly. EMG recordings indicated increased inspiratory activity of the scalene muscles and increased expiratory activity of the transversus abdominis during CPAP, leading to an increase in oxygen consumption from 278 ± 40 ml/min to 322 ± 52 ml/min (p < 0,05) measured with indirect calorimetry.

## Conclusions

In normal individuals 10cm H_2_O of CPAP induces acute overdistention. This overdistension activates the accessory respiratory muscles and increases the work of breathing. The increased inspiratory scalene muscle activity during CPAP explains the increased RC contribution to tidal volume, whereas the expiratory transversus abdominis recruitment probably defends the lung from overinflation by pulling back the diaphragm towards the initial FRC position. We can assume that in patients with acute respiratory failure not tolerating various levels of CPAP, CPAP induces over-distension and increases the work of breathing.

